# Aches and Pains

**Published:** 1875-02

**Authors:** 


### ACHES AND PAINS

MIGHT be very properly spoken of as being from a common cause, congestion of the blood—that is, too much blood in a part where the suffering is. Aches are more properly the result of too much blood in the arteries, and if severe, indicated by throbbing and more or less of a darting, sharp and transient character; if the discomfort is dull, steady, a kind of a hurting, then it is pain, in contradistinction of ache, and arises from there being too much blood in the veins, the ache or pain in either case being caused by too much blood in the arteries or veins, distending them, pressing against the nerve of the part, there being a nerve wherever there is a blood vessel.

Neuralgia is from two Greek words, the first meaning nerve, the other meaning ache; hence, is literally a nerve-ache. Therefore, every ache or pain we have is neuralgia, and the reader will, ever hereafter, have a clear and comprehensive idea of what neuralgia is.

